# Prognostic Factors on Preoperative MRI for Patient-Reported Outcomes After Posterior Medial Meniscus Root Repair

**DOI:** 10.1177/23259671241263648

**Published:** 2024-08-19

**Authors:** Sergio E. Flores, Rawee Manatrakul, Christopher Anigwe, Chotigar Ngarmsrikam, Brian T. Feeley, C. Benjamin Ma, Thomas M. Link, Drew A. Lansdown

**Affiliations:** † Department of Orthopaedic Surgery, University of California, San Francisco, San Francisco, CA, USA; ‡ Department of Radiology, University of California, San Francisco, San Francisco, CA, USA; § Department of Radiology, Faculty of Medicine, Ramathibodi Hospital, Mahidol University, Bangkok, Thailand; Investigation performed at the University of California, San Francisco, San Francisco, California, USA

**Keywords:** knee meniscus, knee articular cartilage, magnetic resonance imaging, patient-reported outcomes

## Abstract

**Background:**

Repair of posterior medial meniscus root (PMMR) tears has demonstrated favorable outcomes and may prevent rapid progression of knee osteoarthritis; however, there is a paucity of data regarding prognostic factors affecting postoperative outcomes.

**Purpose/Hypothesis:**

The purpose of this study was to identify factors on preoperative magnetic resonance imaging (MRI) that predict postoperative outcomes after PMMR repair. It was hypothesized that patients with increasing levels of degenerative changes as evaluated through semiquantitative preoperative MRI scans would have worse postoperative patient-reported outcome (PRO) scores.

**Study Design:**

Cohort study; Level of evidence, 3.

**Methods:**

Patients who underwent PMMR repair between 2012 and 2020 and had minimum 2-year follow-up data were enrolled. Pre- and postoperative visual analog scale pain scores and postoperative PRO surveys including the Patient-Reported Outcomes Measurement Information System–Physical Function, Lysholm knee score, and Knee injury and Osteoarthritis Outcome Score (KOOS) were collected. Patients who achieved the Patient Acceptable Symptom State (PASS) on the KOOS subscales were reported. Two fellowship-trained musculoskeletal radiologists reviewed preoperative MRIs and calculated the Whole-Organ Magnetic Resonance Imaging Score for meniscus, cartilage, bone marrow edema–like lesions (BMELL), and meniscal extrusion. Statistical analysis was performed using the 2-sample *t* test, Mann-Whitney test, and Fisher exact test for categorical variables.

**Results:**

A total of 29 knees in 29 patients were evaluated (22 female, 7 male; mean age at surgery, 52.3 ± 9.9 years; body mass index, 27.6 ± 5.6 kg/m^2^; mean follow-up, 59.6 ± 26.5 months). Visual analog scale for pain scores decreased significantly from preoperatively (4.9 ± 2.0) to final follow-up (1.6 ± 1.9) (*P* < .001), and the percentage of patients meeting the PASS ranged from 44.8% for KOOS Sport and Recreation to 72.4% for KOOS Pain and KOOS Quality of Life. Patients with medial tibial BMELL (MT-BMELL) had significantly lower KOOS Symptoms scores (76.1 ± 17.3 vs 88.4 ± 9.7 without MT-BMELL; *P* = .032). Cartilage quality and presence of meniscal extrusion were not associated with outcomes.

**Conclusion:**

Patients with MT-BMELL on their preoperative MRI in the setting of PMMR tear were found to have worse KOOS Symptoms scores after PMMR repair.

Meniscus root tears are increasingly recognized as significant injuries because of their association with the potential development of rapid knee osteoarthritis.^12^ Approximately 10% to 21% of all knee meniscal tears are root tears.^3,25^ The medial meniscus plays an important biomechanical role in the knee by increasing tibiofemoral contact area and decreasing peak tibiofemoral contact pressures.^1,27^ A cadaveric study determined that posterior medial meniscus root (PMMR) tears had equivalent high peak contact pressure compared with total meniscectomy and that root repair restored these abnormal forces to within normal values.^1^ Due to these stresses, PMMR tears are associated with rapid worsening of cartilage quality and can lead to severe degenerative changes present even within 1 year of the meniscal injury.^26^

Medial meniscus root tears typically occur in knees with cartilage degeneration and older individuals.^24^ Preoperative cartilage quality may play a role in outcomes after repair; patients with significant cartilage degeneration may not be ideal candidates for root repair and may consider arthroplasty, either total or partial, as a surgical treatment option.^30^ The point at which to consider root repair versus arthroplasty treatment options in patients without significant radiographic arthritis is unknown. Other magnetic resonance imaging (MRI) findings, including meniscal extrusion of >3 mm measured on coronal MRI, are commonly observed in medial meniscus root tears; these findings are associated with the development of accelerated knee osteoarthritis, and extrusion may not improve after root repair.^5,12,28^ Extrusion is believed to be associated with greater stress on the medial compartment as well as with cartilage changes.^26,31,37^

PMMR repair with the transtibial pullout repair technique uses fixation through a transtibial tunnel to repair the meniscus root to the anatomic root insertion on the posterior tibia.^8,11,16,19,38^ Multiple studies have shown that medial meniscus root repair, compared with meniscectomy and nonoperative management, leads to fewer patients’ converting to partial or total knee arthroplasty (TKA), less progression of osteoarthritis findings on imaging, and improved patient-reported outcome (PRO) scores.^2,6,7,10,35^ In a matched-cohort study, progression to arthroplasty after nonoperative treatment, partial meniscectomy, and repair was 26%, 60%, and 0%, respectively, at 6-year follow-up.^2^ In a 10-year follow-up study,^6^ progression to TKA was 56% in the partial meniscectomy group and 22% in the repair group, representing a significantly lower conversion rate in those patients with repair, though still not an insignificant group of patients who progressed to TKA after root repair. It is important to identify patients who are at risk of having a poor outcome with root repair to potentially prevent a surgery with a long recovery if they may still need a replacement in the near future.

While we know cartilage defects can play a role in predicting outcomes after PMMR repair, it is unknown how more subtle preoperative MRI findings influence postoperative outcomes.^30^ Semiquantitative MRI can be used for improved detection of degenerative pathology and to calculate the preoperative knee Whole-Organ Magnetic Resonance Imaging Score (WORMS), a validated measure of knee cartilage quality and osteoarthritis.^12,17,32,34^ WORMS evaluation on MRI has been used in past studies to examine overall knee quality in conditions such as osteoarthritis and meniscal tears and can evaluate bone marrow edema–like lesions (BMELL).^12,17,32,34^ BMELL is a term used to describe bone marrow edema patterns or abnormalities in subchondral areas with increased signal, which can be associated with overlying cartilage abnormality, injury, and arthritis.^13,23^o Using preoperative data to provide expected outcomes could allow for informed decision making in the setting of PMMR tear before committing to a root repair surgery with a prolonged rehabilitation and recovery time. Furthermore, if relying on intraoperative cartilage findings to predict outcomes, it limits the option of changing a treatment plan when deciding between arthroscopic options and arthroplasty management.

The purpose of this study was to identify factors on preoperative MRI that predict postoperative outcomes after PMMR repair. We hypothesized that patients with increasing levels of degenerative change as evaluated through semiquantitative preoperative MRI scans would have worse postoperative PRO scores at a minimum 2 years after PMMR repair.

## Methods

### Patient Selection

Patients who underwent PMMR repair at a tertiary referral academic center by 5 fellowship-trained sports medicine surgeons (including C.B.M., B.T.F., D.A.L.) between 2012 and 2020 were retrospectively identified and included in the study. Inclusion criteria included age between 18 and 70 years, PMMR repair through transtibial fixation technique, minimum 2-year postoperative follow-up, and a preoperative knee MRI within 6 months of surgical treatment. Exclusion criteria included prior ipsilateral knee meniscal or ligamentous injury. Institutional review board approval and informed consent were obtained for this study (IRB 21-34760).

### Preoperative Imaging and Imaging Analysis

Preoperative weightbearing anteroposterior fixed-flexion radiographs and 3.0-T MRI were obtained for all patients. The MRI protocol included coronal, sagittal, and axial fat-saturated intermediate-weighted fast spin-echo (FSE) sequences, as well as sagittal proton density–weighted and coronal T1-weighted FSE sequences. Two fellowship-trained musculoskeletal radiologists (R.M., C.N.) reviewed the preoperative MRIs and calculated the WORMS for meniscus, cartilage, and BMELL as well as effusion, synovitis, and meniscal extrusion.^12,28^ A prior study showed high interobserver agreement (intraclass correlation coefficient [ICC] >0.80) for WORMS subsection scoring.^32^ Cartilage was considered normal if WORMS scores were 0 or 1 and abnormal if WORMS scores were 2 to 6. Cartilage degeneration was also assessed via Outerbridge classification as previously described.^33^ If there was a discrepancy with the scoring, a third senior radiologist (T.M.L.) made the final decision on the scoring. Preoperative radiographs were evaluated by the same 2 radiologists to assess the presence and severity of osteoarthritis using the Kellgren-Lawrence (KL) classification.^22^

### Surgical Technique and Postoperative Protocol

A standard knee arthroscopy was performed with lateral and medial portals. The medial meniscus root tear and meniscus were probed and confirmed to be unstable. A standard transtibial pullout meniscus root repair was performed. The root insertion was debrided. Through the medial portal, a suture was passed through the meniscus with a suture passing device. Next, a root repair guide was inserted through the medial portal and placed at the root insertion. A small incision for the drill guide was made on the tibia. A guide pin was drilled, and then a transtibial tunnel was reamed over the pin. The suture from the meniscus was shuttled down the tunnel, placed through a cortical button, and tied to appropriate tension to reduce the meniscus root insertion under direct visualization. The knee was cycled through range of motion, and the meniscus was visualized and probed confirming stability.

Postoperatively, the patients were nonweightbearing for 6 weeks. A hinged knee brace with range of motion between 0° and 90° was used during this time, and patients started a standardized physical therapy rehabilitation program. At 6 weeks after surgery, patients began progressive weightbearing, advanced range of motion as tolerated, and a strengthening regimen. At 4 to 5 months, they were allowed to start a running program and, if involved in sports, were allowed to progressively return to sports at 6 to 9 months once cleared by their treating surgeon.

### Outcome Measures

Visual analog scale (VAS) for pain scores were collected preoperatively and postoperatively. PRO surveys including the Patient-Reported Outcomes Measurement Information System–Physical Function computer adaptive test (PROMIS-PF CAT), Lysholm knee score, and Knee injury and Osteoarthritis Outcome Score (KOOS; divided into 5 subscales: Symptoms, Pain, Activities of Daily Living [ADL], Sport and Recreation [Sport/Rec], and Quality of Life [QoL]) were collected at minimum 2 years postoperatively.^9,10,21,35,36^ In addition, we identified the patients who met the Patient Acceptable Symptom State (PASS) for the KOOS subscales using PASS values from a prior meniscal repair study.^29^ In the orthopaedic surgery literature, there has been a growing interest in PROMIS scores because they can be compared across various studies, they are efficiently administered as a CAT, and they correlate well with legacy measures.^4^ For meniscus root tears, preoperative PROMIS-PF CAT scores have correlated well with the KOOS, with no floor or ceiling effects and requiring as few as 4 questions to administer.^14,15^

### Statistical Analysis

Statistical analysis was performed with Stata 16.1 (StataCorp) using the 2-sample *t* test, Mann-Whitney test, and Fisher exact test for categorical variables. The Spearman rank correlation was utilized to test for relationships between continuous variables. Significance was defined as *P* < .05. A power analysis was performed that determined that a correlation of 0.50 with 80% power and alpha of .05, 29 patients would need to be enrolled.

## Results

There were 56 patients with 58 knees eligible for the study. Nine patients declined participation, 1 was deceased, and 19 patients could not be reached by email and phone after attempting contact. Of those eligible patients, 2 converted to arthroplasty (1 partial, 1 total), but they did not consent to participate in the study. No patients required a subsequent arthroscopy procedure. The final study cohort included 29 knees in 29 patients (22 female, 7 male) with a mean age at surgery of 52.3 ± 9.9 years and body mass index of 27.6 ± 5.6 kg/m^2^, evaluated at a mean follow-up of 59.6 ± 26.5 months (Table 1).

**Table 1:** Patient and Injury Characteristics^*a*^

Variable	Value
Sex, n	
Female	22
Male	7
Age, y, mean ± SD	52.3 ± 9.9
BMI, kg/m^2^, mean ± SD	27.6 ± 5.6
Laterality, n	
Right	13
Left	16
Follow up, mo, mean ± SD	59.6 ± 26.5
KL score, median	1
Time from symptoms to surgery, d, mean ± SD	112.3 ± 73.6

a BMI, body mass index; KL, Kellgren-Lawrence.

**Figure 1.**
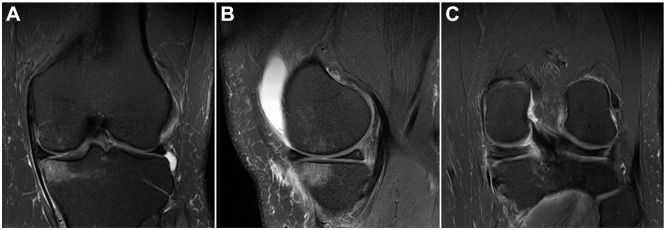
T2-weighted magnetic resonance imaging scans. (A) Coronal and (B) sagittal images showing a medial tibial bone marrow edema–like lesion. (C) Coronal image demonstrating a posterior medial meniscus root tear.

Osteoarthritis ranged from KL grades 0 to 2, with 79% KL grades 0 to 1 and 21% grade 2. Five patients either did not have preoperative radiographs accessible for review or they were not yet weightbearing. Coronal intermediate-weighted FSE images showed meniscal extrusion in 14 of 29 (48.3%) of patients with a mean extrusion of 4.1 ± 0.70 mm. WORMS values for abnormal cartilage and BMELL based on knee region are shown in Table 2. Results of cartilage assessments are also shown in Table 2, displaying knees with Outerbridge scores 0 to 2 versus 3 to 4. Cartilage was most commonly abnormal in the medial femoral condyle, trochlea, and patella. BMELL was most commonly found in the medial femoral condyle, medial tibia, and patella. An example of medial tibial BMELL (MT-BMELL) is shown in Figure 1. The ICCs regarding BMELL scoring between the 2 radiologists were >0.8 for the patella, trochlea, medial compartment, and lateral compartment, indicating high interobserver agreement.

**Table 2:** Results of Preoperative Imaging Analysis^*a*^

	MFC (n)	MT (n)	LFC (n)	LT (n)	Trochlea (n)	Patella (n)
Cartilage						
WORMS 0-1 (normal)	12	21	24	22	15	8
WORMS 2-6 (abnormal)	17	8	5	7	14	21
Outerbridge grades 0-2	19	26	24	28	19	14
Outerbridge grades 3-4	10	3	5	1	10	15
Bone marrow edema						
BMELL absent	19	16	28	28	22	18
BMELL present	10	13	1	1	7	11

a Values presented as number of knees with finding. BMELL, bone marrow edema–like lesion; LFC, lateral femoral condyle; LT, lateral tibia; MFC, medial femoral condyle; MT, medial tibia; WORMS, Whole-Organ Magnetic Resonance Imaging Score.

VAS for pain scores decreased significantly from before surgery to final follow-up (4.9 ± 2.0 vs 1.6 ± 1.9, respectively; *P* < .001), and the percentage of patients meeting PASS ranged between 44.8% for the KOOS Sport/Rec subscale and 72.4% for KOOS Pain and KOOS QoL subscales (Table 3). Mean postoperative PRO scores are also listed in Table 3.

**Table 3:** Patient Reported Outcome Scores^*a*^

Outcome Measure	Score, Mean ± SD	Achieved PASS, %
VAS pain		
Preoperative	4.9 ± 2.0	—
Postoperative	1.6 ± 1.9	—
PROMIS-PF CAT	50.6 ± 6.1	—
Lysholm knee score	83.1 ± 19.4	—
KOOS Symptoms	82.9 ± 14.7	65.5
KOOS Pain	87.3 ± 13.4	72.4
KOOS ADL	91.0 ± 13.7	58.6
KOOS Sport/Rec	77.1 ± 23.4	44.8
KOOS QoL	70.5 ± 25.7	72.4

a ADL, Activities of Daily Living; CAT, computer adaptive test; KOOS, Knee injury and Osteoarthritis Outcome Score; PROMIS-PF, Patient-Reported Outcomes Measurement Information System–Physical Function; QoL, Quality of Life; Sport/Rec, Sport and Recreation; VAS, visual analog scale. Dashes indicate areas not applicable.

**Figure 2.**
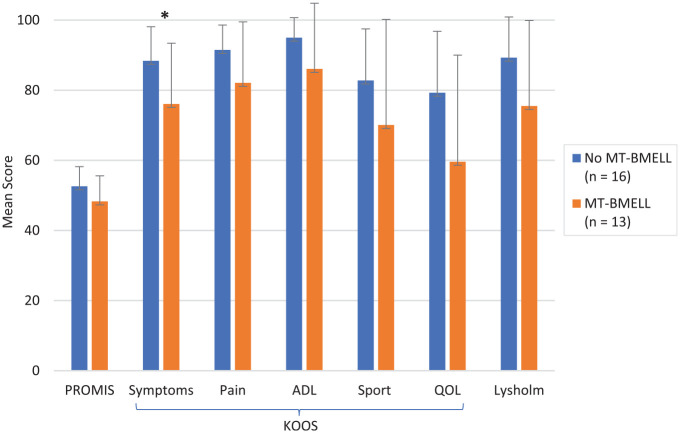
Postoperative mean patient-reported outcome scores between patients with and without MT-BMELL on preoperative magnetic resonance imaging. Error bars represent standard deviation. *Statistically significant difference between MT-BMELL and no MT-BMELL (*P* < .05). MT-BELL, medial tibial bone marrow edema–like lesions; ADL, Activities of Daily Living; KOOS, Knee injury and Osteoarthritis Outcome Score; PROMIS, Patient–Reported Outcomes Measurement Information System; QoL, Quality of Life; Sport, Sport and Recreation.

There were 13 patients with MT-BMELL on preoperative MRI and 16 patients with no medial tibial BMELL. When comparing patients with versus without medial tibial BMELL, those with medial tibial BMELL had significantly lower KOOS Symptoms scores (76.1 ± 17.3 vs 88.4 ± 9.7 *P* = .032) and lower but nonsignificant scores for the PROMIS-PF CAT (48.3 ± 7.3 vs 52.6 ± 5.6; *P* = .087), Lysholm (75.5 ± 24.4 vs 89.3 ± 11.6; *P* = .16), KOOS Pain (82.1 ± 17.4 vs 91.5 ± 5.7; *P* = .20), KOOS ADL (86.1 ± 18.7 vs 95.0 ± 5.7; *P* = .33), KOOS Sport/Rec (70.1 ± 30.1 vs 82.8 ± 14.7; *P* = .28), and KOOS QoL (59.6 ± 30.4 vs 79.3 ± 17.5; *P* = .068) (Figure 2). Table 4 shows the percentage of patients meeting the PASS stratified according to presence of MT-BMELL. In patients with medial tibial BMELL, the time from reported symptoms to MRI ranged from 1 to 188 days, with a mean of 60 days. The time to surgery for those with versus without medial tibial BMELL was not significantly different (50.2 ± 57.4 vs 68.25 ± 63.1 days, respectively; *P* = .53). There were no significant differences in patient characteristics between those with and without MT-BMELL. There was no significant difference in PRO scores when comparing those with BMELL in any additional knee region, cartilage quality in any of the knee regions, presence of meniscal extrusion, or presence of edema at the meniscus root insertion. There was no significant association between cartilage WORMS scores and PRO scores. There were no significant differences in PRO scores comparing Outerbridge grades 0 to 2 versus grades 3 to 4 (*P* > .05 for all). Additionally, no significant association was found between PRO outcomes when examining, time to injury, time to symptoms, and time to surgery, nor time from MRI to surgery.

**Table 4:** Percentage of Patients Who Met the PASS for KOOS Subscales According to MT-BMELL Presence^*a*^

	PASS, % (n)		
Outcome Measure	MT-BMELL Present, n = 13	MT-BMELL Absent, n = 16	*P* ^*b*^
KOOS Symptoms	46.2 (6)	81.3 (13)	.064
KOOS Pain	53.8 (7)	87.5 (14)	.092
KOOS ADL	53.8 (7)	62.5 (10)	.72
KOOS Sport/Rec	46.2 (6)	43.8 (7)	≥.999
KOOS QoL	53.8 (7)	87.5 (14)	.092

a ADL, Activities of Daily Living; KOOS, Knee injury and Osteoarthritis Outcome Score; MT-BMELL, medial tibial bone marrow edema–like lesion; PASS, Patient Acceptable Symptom State; QoL, Quality of Life; Sport/Rec, Sport and Recreation.

b Fisher exact test.

## Discussion

The study findings showed that patients with MT-BMELL on their preoperative MRI had significantly inferior KOOS Symptoms scores after PMMR repair compared with those without medial tibial BMELL. The goal of this study was to identify patients who would have worse PRO scores after surgery based on preoperative radiographs and MRI. In this cohort, pain scores significantly improved postoperatively and >70% of patients reached PASS for KOOS Pain and KOOS QoL. The postoperative Lysholm scores were similar to those reported in the PMMR repair studies by Moon et al^30^ and Kim et al.^20^ However, for scores that did not reach statistical significance, including the PROMIS-PF, these improvements may not be clinically significant.

Patients with PMMR tears are typically older and female and show signs of cartilage degeneration.^24^ These demographics were similar to our study patients, who had a mean age of 52.3 years and a median KL grade of 1, and included many patients with degenerative cartilage changes on MRI. In our study, cartilage quality on preoperative MRI was not significantly associated with patient outcomes. Moon et al^30^ found that modified Outerbridge grade 3 or 4 chondral lesions on the weightbearing portions of the medial compartment on preoperative MRI was an independent risk factor for unfavorable clinical outcomes after root repair, but the presence of subchondral edema was not related. One explanation as to the difference in results may be that Moon et al^31^ had a higher rate of patients with medial compartment focal Outerbridge grade 3 or 4 (51% compared with 34% in our cohort). Medial tibial cartilage is often thin on MRI, and cartilage changes can be difficult to identify; therefore, bone marrow lesions could be a marker of subtle cartilage injury. There was a higher rate of PASS achievement for patients without MT-BMELL for KOOS subscales except for KOOS Sport/Rec; however, these comparisons were not statistically significant. In patients with MT-BMELL, the time from reported symptoms to MRI was a mean of 60 days. Therefore, it is difficult to know whether BMELL stems from the medial meniscus root tear injury or is a result of a chronic degenerative process in the knee, which could point to why those with the presence of this finding may have worse outcomes. Previous studies have shown that repair may not decrease meniscal extrusion; however, early repair before 13 weeks may minimize progression of extrusion.^18,30,31^ We did not examine progression of meniscal extrusion in this study; however, there was no correlation with preoperative presence of extrusion and patient outcomes.

Although most patients have improved outcomes and decreased conversion to arthroplasty after medial meniscus root repair, there are still patients with poor PRO scores or progress to arthroplasty.^6,10^ Based on prior studies by Bernard et al^2^ and Chung et al^6^, patients who undergo PMMR compared with meniscectomy or nonoperative treatment have better outcomes and decreased progression to TKA at 6- and 10-year follow-up, respectively. Patients may be excluded from surgery based on surgeon judgment if they have large obvious cartilage defects, but this may fail to take into consideration patients with more subtle overall knee pathology such as BMELL, which could point to a more chronic degenerative process in the knee. Successful outcomes after surgery are also likely to depend upon close adherence to an appropriate rehabilitation program, including a prolonged period of nonweightbearing on the affected leg that may be especially challenging for older patients.

The decision to proceed with PMMR repair versus nonoperative treatment or arthroplasty depends on many factors the surgeon could take into consideration including age, function, and pre-existing cartilage changes. We found that patients with MT-BMELL had worse PRO outcomes; however, PMMR repair improved outcomes and many patients met PASS for the KOOS subscales overall. There was a relatively low rate of meeting the PASS for KOOS Sport/Rec and KOOS ADL in patients with and without MT-BMELL. One explanation could be that the PASS threshold of 80 points for KOOS Sport/Rec and 92.7 points for KOOS ADL used in this study were extrapolated based on outcomes after arthroscopic meniscal repair,^29^ which may be a different population than those with root tears. We do believe that even with presence of MT-BMELL and mild cartilage degeneration, surgeons should still offer PMMR to most patients and can counsel them regarding expectations and potentially worse outcomes. We do not know if the patients in this cohort with MT-BMELL have a higher chance of converting to knee arthroplasty; this could be a potential future study. Considerations to treating nonoperatively or planning toward arthroplasty may be more appropriate if the patient is older and lower functioning or cannot adhere to nonweightbearing and physical therapy protocols.

### Limitations and Strengths

Limitations of this study include the fact that only the VAS for pain score was measured preoperatively, and there was no analysis of postoperative imaging. We acknowledge that without preoperative PRO scores we may be missing a change in the scores; however, we had similar results to previous studies in terms of postoperative PRO scores. Although we did not have postoperative MRI images, the goal was to identify preoperative MRI findings that could predict postoperative clinical scores, not to track and associate the changes seen on postoperative MRI. Given the retrospective nature of the study, we lost eligible patients to follow-up and thus were unable to evaluate their MRI or obtain PRO scores. Although conversion to knee arthroplasty was not an exclusion criterion, the 2 eligible study patients who converted did not consent to the study; therefore, we could not evaluate their preoperative MRI data for MT-BMELL. Multivariate analysis including KL grade was not possible due to the relatively small sample size of the series and limited follow-up rates which is a limitation to this study. However, we found that there were no demographic differences in the patients with and without MT-BMELL. This study may also be underpowered to detect associations between cartilage and PRO scores. Finally, in this retrospective study, the transtibial surgical technique used for root repair was similar in all patients, but there may have been some variability in individual surgeon technique. A strength of this study is the mean follow-up of approximately 5 years and preoperative MRI data on all patients. Furthermore, this is the first study to our knowledge that evaluated PROMIS-PF scores and patients meeting PASS for KOOS subscales after PMMR repair.

## Conclusion

The presence of MT-BMELL on preoperative MRI was found to be a marker for inferior patient outcomes for KOOS Symptoms scores at minimum 2 years after PMMR repair.
